# Do gender differences in primary PCI mortality represent a different adherence to guideline recommended therapy? a multicenter observation

**DOI:** 10.1186/1471-2261-14-71

**Published:** 2014-06-02

**Authors:** Ralf Birkemeyer, Henrik Schneider, Andreas Rillig, Juliane Ebeling, Ibrahim Akin, Stefan Kische, Liliya Paranskaya, Werner Jung, Hueseyin Ince, Christoph A Nienaber

**Affiliations:** 1 Department of Cardiology, Heart Center Rostock, Medizinische Klinik I, Universitätsklinikum Rostock, Ernst-Heydemann-Str. 6, 18057 Rostock, Germany; 2 Department of Cardiology, Asklepios Klinik St. Georg, Hamburg, Germany; 3 Department of Cardiology, Schwarzwald-Baar-Klinikum, Villingen-Schwenningen, Germany

**Keywords:** Primary PCI, Myocardial infarction network, Gender differences

## Abstract

**Background:**

It is uncertain whether gender differences in outcome after primary percutaneous coronary intervention (PCI) are only attributable to different baseline characteristics or additional factors.

**Methods:**

Databases of two German myocardial infarction network registries were combined with a total of 1104 consecutive patients admitted with acute ST-elevation myocardial infarction (STEMI) and treated according to standardized protocols.

**Results:**

Approximately 25% of patients were females. Mean age (69 vs 61 years), incidence of diabetes (28% vs 20%), hypertension (68 vs 58%) and renal insufficiency (26% vs 19%) was significantly higher compared to males. Mean prehospital delay was numerically longer in females (227 vs 209 min) as was in hospital delay (35 vs 30 min). PCI was finally performed in 92% of females and 95% of males with comparable procedural success (95% vs 97%). Use of drug eluting stents (55% vs 68%) and application of GP 2b 3a blockers (75% vs 89%) was significantly less frequent in women. At discharge, prescription of beta blockers and lipid lowering drugs was also significantly lower in females (84% vs 90% and 71% vs 84%). Unadjusted in-hospital mortality was significantly higher in females (10% vs 5%) without attenuation after 12 months. Adjusted mortality however did not differ significantly between genders.

**Conclusion:**

Higher unadjusted mortality in females after primary PCI was accompanied by significant differences in baseline characteristics, interventional approach and secondary prophylaxis in spite of the same standard of care. Lower guideline adherence seems to be less gender specific but rather a manifestation of the risk-treatment paradox.

## Background

Gender differences in outcome after acute ST-elevation myocardial infarction (STEMI) are well known [1-5]. This holds true irrespective of performance of mechanical reperfusion with primary percutaneous coronary intervention (PCI) in most large observations [6,7], although there are single contradictory reports [8]. There is some uncertainty whether higher mortality in females is only attributable to different baseline characteristics or additional factors such as delayed diagnosis and reperfusion, under-treatment or genuine gender specific differences in therapeutic susceptibility [1-9]. Female STEMI patients usually present at higher mean age than males and therefore at higher risk. Interactions between higher risk and less intensive treatment, the risk-treatment paradox, have been described [10-12].

Our objective was to compare indicators of guideline adherent therapy in a large cohort of consecutive STEMI patients according to gender in the defined setting of a myocardial infarction network aiming at primary PCI for all STEMI patients according to the same treatment algorithm. For this purpose we combined data from two German myocardial infarction network registries. A previous observation in one of the networks had shown that more than 90% of the regional STEMI population received primary PCI after network implementation. In the cohort of patients who did not receive any revascularization attempt, mainly because of age and comorbidities, the proportion of females was reduced from 77% to 44% [13].

## Methods

### Network structures

Both networks aim at reperfusion therapy with primary PCI for all regional STEMI patients according to a uniform, regional treatment protocol during 24h/7d a week in one interventional centre.

Network A is located in the North-eastern Germany and comprises both an urban and a rural catchment area with a population of approximately 415.000 inhabitants. The diameter of the network area is up to 120 km. At the time of data collection there were eight hospitals in the network area, seven of them without cathlabs and one with a high-volume interventional facility and a 24h/7d primary PCI service. Emergency medical services (EMS) transferred STEMI patients to the nearest hospital without announcement. After admission local emergency departments alarmed the interventional team and organized direct transfer to the cathlab.

Network B is located in the South-western Germany and comprises a rural catchment area with a population of approximately 350.000 inhabitants. The diameter of the network area is up to 70 km. At the time of data collection there were six hospitals in the network area, five of them without cathlabs and one with a high-volume interventional facility and a 24h/7d primary PCI service. Network structures included 12-lead ECG in the ambulance, ECG telemetry to the intensive care unit of the invasive facility, a structured phone call between EMS and the intensive care physician on call and preparation of the cathlab before patient arrival. STEMI patients were intended to be directly admitted to the cathlab, irrespective of the presence of cardiogenic shock or resuscitation.

### Primary PCI protocol

All patients were treated with 250–500 mg aspirin intravenously and received a weight adjusted unfractionated heparin dose of 70 IU/kg by EMS or the emergency department. A clopidogrel loading dose of 600 mg was administered either by EMS or the emergency department in most cases. Otherwise, it was given directly before or immediately after the intervention. Operators of patients in shock were encouraged to treat all presumably hemodynamically relevant non-target lesions. Thrombectomy, periprocedural GP2b3a blockers (predominantly abciximab) and drug eluting stents were used at the discretion of the operator. Full dose anticoagulation with heparin was stopped after PCI, unless there was a high thromboembolic risk (e.g. atrial fibrillation or mechanical heart valves).

### Study population

Consecutive STEMI patients admitted for primary PCI were prospectively included in their respective registries, in network A from 2001 to 2003 (n = 603) and in network B from 2005 to 2007 (n = 501).

### Definitions

The diagnosis of ST-elevation acute myocardial infarction was based on the presence of chest pain lasting > 20 min and of significant ST-segment elevation (>0.1 mV in two adjacent leads if leads I-III, aVF, aVL, V4-V6, and ≥ 0.2 mV in leads V1-V3), as recorded in the first ECG obtained. Patients with persistent angina and presumably new left bundle branch block were included in the registry if myocardial infarction was subsequently confirmed. Cardiogenic shock was defined clinically by the presence of hypotension (systolic blood pressure < 90 mm Hg for ≥30 minutes or need for vasopressors to maintain systolic blood pressure >90 mm Hg) and tachycardia (heart rate >90 beats/min) with evidence of end-organ hypo-perfusion [14]. Thrombolysis In Myocardial Infarction (TIMI) flow grades were assessed in the culprit vessel before and after the PCI procedure. No reflow was defined as TIMI 0 flow after successful interventional treatment of the culprit lesion.

Major bleeding was defined according to the TIMI major bleeding definition as intracerebral bleeding, bleeding requiring surgical intervention, bleeding requiring transfusion or loss of more than 5 g% haemoglobin [15].

As indicators of guideline adherent therapy we analysed pre- and in-hospital delays, procedural success of primary PCI, stent use, peri-interventional antiplatelet management, medication at discharge and medication at 12 months [16]. Procedural success was defined as residual stenosis < 30% of the culprit lesion.

For outcomes we analysed mortality, re-infarction rate, TLR and TVR until 12 month.

### Data collection and follow-up

All patients were prospectively documented in a dedicated database. Follow-up was obtained from telephone interviews and questionnaires at 6 and 12 months. Complete follow-up concerning mortality was obtained from state registries.

The registry was approved by the Freiburg Ethics Commission International. All patients were asked for their written informed consent for the extension of our routine follow-up.

### Statistical methods

Data was analyzed according to established standards of descriptive statistics. Categorical variables were compared by χ^2^ test. Continuous variables are reported as mean ± standard deviation or median with interquartile ranges. For comparisons, the *t* test or the two-tailed Mann–Whitney *U* test was used as appropriate. Odds ratios and 95% confidence intervals were provided where appropriate. A *p* value of less than 0.05 was considered significant.

A multivariate logistic regression analysis (stepwise forward model) with gender as a fixed parameter was performed to determine independent factors predicting 12-month mortality. The following 6 variables were identified: age, beta-blocker medication at discharge, diabetes, lipid lowering medication at discharge, shock and renal impairment. The logistic model showed a good predictive value (C-statistic = 0.85), and good calibration characteristics using the Hosmer-Lemeshow test (p = 0.78).

Mortality at 12 months was adjusted for covariates and for propensity score alone, as well as for the covariates with propensity score added as an additional covariate.

## Results

One thousand one hundred and four consecutive patients (n = 1104) with the diagnosis of acute STEMI were prospectively included in the combined registries: 281 women and 823 men.

Mean age (69 vs 61 years, p < 0.01), incidence of diabetes (28% vs 20%, p < 0.01), hypertension (68% vs 58%, p < 0.01) and renal insufficiency (26% vs 19%, p < 0.01) was significantly higher in females compared to males. However, significantly more males were smokers (23% vs 46%, p < 0.01). 9% of patients in both groups were in cardiogenic shock. 6% of females and 9% of males were admitted after resuscitation (p = 0.2) (Table 1).

**Table 1:** Baseline clinical characteristics according to gender

	**Women (n = 281)**	**Men (n = 823)**	**p value**
Age (yrs.)	69 ± 11	61 ± 12	< 0.01
Diabetes	28%	20%	< 0.01
Current smoker	23%	46%	< 0.01
Arterial hypertension	68%	58%	< 0.01
Hyperlipidemia	41%	46%	0.21
Creatinine clearance < 60 ml/min	26%	19%	< 0.01
Previous myocardial infarction	7%	11%	0.12
Previous PCI	5%	8%	0.06
Peripheral artery disease	3%	5%	0.31
Previous TIA/stroke	7%	4%	0.09
Cardiogenic shock	10%	10%	0.96
Post CPR	6%	9%	0.15
Systolic blood presssure	134 ± 3	131 ± 1	0.14
Diastolic blood pressure	74 ± 2	75 ± 1	0.77
Heart rate	81 ± 2	76 ± 1	<0.01

Data presented as mean value ± SD or percentage of patients.

CPR: cardiopulmonary resuscitation, PCI: percutaneous coronary intervention.

Mean transfer distances were nearly identical in both groups (21 km). EMS escorted 55% and 56% of female and male patients as announced STEMI to the primary PCI centre. Mean pre-hospital delay was insignificantly longer in females (227 vs 209 min, p = 0.2) as was in-hospital delay (35 vs 30 min, p = 0.4). PCI was finally performed in 92% of females and 95% of males (p = 0.1) with comparable procedural success (95% vs 97%, p = 0.1). Use of drug eluting stents (55% vs 68%, p = 0.03) and application of GP2b3a blockers (75% vs 89%, p < 0.01) was significantly less frequent in women (Table 2).

**Table 2:** Reperfusion delays and primary PCI details according to gender

	**Women (n = 281)**	**Men (n = 823)**	**p value**
Pre-hospital delay (min)*	169 (104;296)	158 (90;278)	0.08
In-hospital delay (min)*	18 (7;39)	15 (6,31)	0.07
Announced EMS escorted transfer	55%	56%	0,75
No coronary artery stenosis > 50%	1%	2%	0.97
Multivessel disease	48%	48%	0.97
Culprit vessel LAD	41%	43%	0.70
Culprit vessel LMS	1%	0%	0,70
PCI performed	92%	95%	0.07
Multivessel PCI performed	5%	4%	0,80
Further staged PCI	19%	21%	0,51
Staged CABG	1%	3%	0,12
Average number of stents implanted^§^	1.41	1.45	0,69
Stent length (mm)^§^	29.2	30.9	0.36
Minimal stent diameter (mm)^§^	2.9	3.2	0.15
Drug eluting stent	55%	68%	0,03
Peri- or intraprocedural GP2b3a blocker	75%	89%	<0.01
Residual diameter stenosis after PCI < 30%	95%	97%	0.12
Pre-procedural TIMI 0/3 flow^§^	60%/17%	60%/17%	0.99
Post-procedural TIMI 0/3 flow^§^	6%/81%	7%/82%	0,23

*Data analysed in the 216 females and 708 males where symptom onset could be clearly assigned to the 12h period before hospital admission; pre-hospital delay: symptom onset until admission to interventional hospital; in-hopital delay: admission until start of angiography.

^§^Complete data only available from network B.

Data presented as median with interquartile ranges or percentage of patients.

CABG; coronary artery bypass grafting; EMS: emergency medical services; LAD: left anterior descendant; LMS: left main stem; PCI: percutaneous coronary intervention.

In spite of lesser use of GP2b3a blockers major bleeding was encountered significantly more often in females (6% vs 2%; p < 0.01) (Table 3).

**Table 3:** In-hospital bleeding complications according to gender

	**Women (n = 281)**	**Men (n = 823)**	**P value**
Major bleeding*	6%	2%	< 0.01.
Minor bleeding^§^	4%	7%	0.22
Insignificant bleeding^$^	19%	14%	0.21

Data presented as percentage of patients.

*intra-cerebral bleeding, bleeding requiring surgical intervention or transfusion, loss of haemoglobin ≥ 5g%; ^§^haematuria, haematemesis, loss of haemoglobin ≥ 3g% and <5 g% with or ≥ 4g% and < 5g% without identifiable source of bleeding; ^$^bleedings not fulfilling the criteria of a major or minor bleeding.

At discharge prescription of beta-blockers and lipid lowering drugs was also significantly lower in females (84% vs 90%, p < 0.01 rsp. 71% vs 84%; p < 0.01). These differences were more pronounced in network A. Numerical differences in prescription persisted at 12 months (data only available for network B) (Table 4).

**Table 4:** Medication at discharge and 12 months according to gender

	**At discharge**			At 12 months*		
	**Women**	**Men**	**p value**	**Women**	**Men**	**p value**
	**(n = 253)**	**(n = 795)**		**(n = 97)**	**(n = 375)**	
ASS	91%	94%	0.05	56%	65%	0.07
Clopidogrel	88%	93%	0.02	31%	40%	0.10
Anticoagulation	10%	9%	0.80	8%	5%	0.20.
Triple therapy	8%	8%	0.69	0%	2%	0.20.
Beta-Blocker	84%	90%	<0.01	51%	61%	0.06
ACE inhibitor	76%	79%	0.18	50%	58%	0.15
Lipid lowering drug	71%	84%	<0.01	53%	61%	0.14

Data presented as percentage of patients.

*12-month data only available for network B.

**Figure 1:**
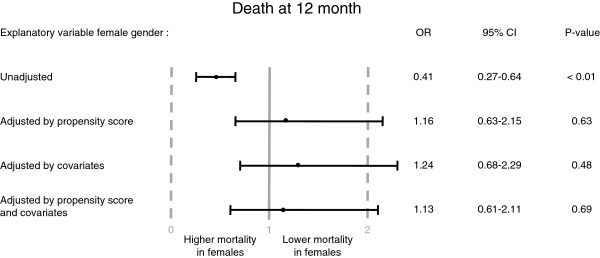
**Death at 12 months.** Explanatory variable female gender: unadjusted, adjusted by propensity score, covariates or by covariates with propensity score as additional covariate (details under “Statistical methods”).

Unadjusted in-hospital mortality was significantly higher in females (10% vs 5%, p < 0.01). Difference persisted during the first year without attenuation (15% vs 7%, p < 0.01). Re-infarction, target lesion revascularisation and target vessel revascularisation rates were numerically lower in females within the first year after the index event (Table 5).However, after adjustment by propensity score or covariates, female gender failed to be predictive as explanatory variable for 12 month mortality (Figure 1).

**Table 5:** Major adverse cardiac events until 12 months according to gender

	**Women**	**Men**	**p value**
Mortality:			
In-hospital	10.0%	4.5%	< 0.01
6-month	14.2%	6.9%	< 0.01
12-month	14.9%	6.9%	<0-01
STEMI*	1.9%	3.8%	0.31
NSTEMI*	1.9%	2.0%	0.90
Clinically driven TLR*	3,7%	6.6%	0.28
Clinically driven TVR*	5.6%	7.4%	0.54
Definite stent thrombosis*^§^	0.9%	2.3%	0.46

Data presented as percentage of patients.

NSTEMI: non ST-elevation myocardial infarction; STEMI: ST-elevation myocardial infarction; TLR: target lesion revascularisation; TVR: target vessel revascularisation.

*Complete data only available from network B.

^§^ARC definition.

## Discussion

The analysis of 1104 consecutive STEMI patients admitted for primary PCI according to the uniform standard of care of a myocardial infarction network, showed that early and late mortality in females was more than double the mortality of males. This was accompanied by significant differences in baseline characteristics as has been described in a number of previous observations [1-5]. Mean age of women presenting with STEMI was approximately eight years older compared to men. Furthermore incidence of diabetes, hypertension and renal impairment was significantly higher in the female cohort.

Comparable gender differences in mortality have been found in large registries [1-5]. Single observations reported, however, similar unadjusted mortality of males and females after primary PCI [8]. This might be due to different patient selection.

Under network conditions more than 90% of all regional STEMI patients are treated with primary PCI rendering the analysis highly representative for an unselected STEMI population suitable for revascularisation. We have shown that in the remaining subset of predominantly elderly patients who are not scheduled for any revascularisation attempt females were not overrepresented [13].

A unisex standard of care for STEMI patients reflects current guidelines [16,17] with primary PCI being the preferred reperfusion strategy for both genders. It has been demonstrated that primary PCI is equally effective in men and women [18,19].

Although standard of care in the network regions was not gender specific, actual treatment showed relevant differences. There was a trend to more frequent abortion of intended PCI in females, although the proportion of patients without significant coronary artery disease and patients with multi-vessel disease was quite comparable in both cohorts. In contrast to our finding, a significantly higher proportion of non-obstructive coronary artery disease has been previously described in female compared to male ACS patients in a large meta-analysis [1]; this difference was however smallest in the subset of ACS patients presenting with STEMI. The use of drug eluting stents and GP2b3a blockers was significantly lower in females whereas pre- and in-hospital delays before primary PCI were only numerically longer and the immediate result of primary PCI comparable. Reduction of system related time delays before primary PCI is obviously a primary objective of myocardial infarction networks and has been successfully proven [13,20-23]. Attempts to intervene on patient related time delays showed no similar success [24]. A comparable pattern of slightly increased system delays and lesser use of drug eluting stents and GP blockers seen in females has also been shown in elderly network patients [25].

More strikingly, was the significantly lower prescription of beta-blockers and lipid lowering drugs as well as the numerically lower prescription of anti-platelets in females at discharge. The differences in anti-platelet medication cannot be fully explained by the slight differences in the need for anticoagulation, but rather by the significantly higher incidence of major bleedings in females during hospitalisation in spite of less aggressive peri-interventional platelet management. Higher bleeding rates after primary percutaneous intervention in females have been described in many observations [26-28]. The lesser prescription of betablockers in females cannot explained by lower blood pressure or heart rate on admission. Numerical differences in recommended secondary prophylaxis persisted over 12 months. Thus adherence to guideline recommended therapy was lower in females than males. Again, this pattern of less guideline adherent secondary prophylaxis in females has also been observed in elderly patients [25].

Multivariate analysis suggested that both the different baseline characteristics as well as the lesser use of recommended secondary prophylaxis had an independent influence on mortality. Interestingly, female gender failed to be predictive as explanatory variable for mortality after adjustment by propensity score or covariates. So there was no implication of gender differences in susceptibility to primary PCI or of a gender specific general under use of therapy in this analysis. The observed differences in guideline adherence with respect to secondary prophylaxis might be more related to the different baseline characteristics which clearly attributed a higher risk to females.

The risk-treatment paradox has been previously described [10-12]. Actually, myocardial infarction networks counteract this paradox by aiming at higher reperfusion rates also in high risk patients (especially the elderly and shock patients) [13,20-23]. This raises the question if the reluctant use of recommended secondary pharmacological prophylaxis in the subset of high risk elderly women reflects a residual under use of justified therapy or a truly higher prevalence of contra-indications to this therapy. At least with respect to antiplatelet therapy, increased bleeding risk seemed to be a limiting factor.

Our data also confirmed the previous description of a lower target lesion and target vessel revascularisation rate during long term follow up in females compared to males [29]. It is however questionable if this is primarily attributable to gender specific biological reactions as this phenomenon was also described for the comparison of octogenarians with younger patients after stenting in an all-comer population [30].

### Limitations

A major limitation is the lack of external monitoring of the registries which is an inherent weakness of many investigator-driven observational studies.

A further limitation of our registry is that we only included primary PCI patients and not all regional STEMI patients. Therefore our analysis cannot be extended to the complete STEMI population. Preceding analyses, however, showed that in the setting of a myocardial infarction network more than 90% of the STEMI population were scheduled for mechanical reperfusion.

## Conclusions

Higher unadjusted mortality in females after primary PCI was accompanied by significant differences in baseline characteristics, interventional approach and secondary prophylaxis in spite of a gender neutral standard of care which enabled similar reperfusion rates and attenuation of time delays before primary PCI. Lower guideline-adherence in females in this setting seemed to reflect predominantly a residual risk-treatment paradox and not a gender specific under-treatment. Adjusted mortality showed a favourable trend for females.

## Competing interests

The authors declare that they have no competing interests.

## Authors’ contributions

RB, HS, AR, IA, SK, LP, WJ, HI and CAN participated in treating the patients in the cathlab/ICU and in acquisition of data. RB and JE performed the statistical analysis. RB, HS and CAN drafted the manuscript. All authors read and approved the final manuscript.

## Pre-publication history

The pre-publication history for this paper can be accessed here:

http://www.biomedcentral.com/1471-2261/14/71/prepub
